# 

**Published:** 1889-05

**Authors:** 


### TABLE OF CONTENTS.

ORIGINAL AND SELECTED.
Charge to the Graduates in Medicine .......... 161
Charge to the Graduates in Dentistry .... .... 165
The transplanting of a rabbit's cornea into the human eye .......... 167
A case of Opium Narcosis, &c., .......... 170
A case of transfusion of blood .......... 172
Creolin in Gonorrhœa .......... 173
A new method of operating for the restoration of the female perineum .......... 174
ABSTRACTS &c.
Mr. Fleming, M. R. C. S., says; Hernia; How Vaccination takes with the Indians .......... 179
Treatment of Hemorrhagic Malarial Fever..... 177
Cocaine in Detention .......... 181
How to preserve the Physician's hands; Some of the abuses of Etherization .......... 182
Sulphonal; Magnesii Salicylas .......... 183
Ulexine; Kandol; How to distinguish antifebrin from phenacetin .......... 184
Contraindications for the use of antipyrin, &c.; Gonorrhœal Rheumatism .......... 185
Treatment of Baldness; A new treatment of erysipelas .......... 186
Urine of Business men; Perforative peritonitis, &c. .......... 187
On the treatment of the morphine Habit.... ... 188
Treatment of Ingrowing toe-nail .......... 189
Emptying the bladder by manual compression; Treatment of Dysentery &c. .......... 190
SCIENTIFIC.
Whistling speech; The Gatling Gun .......... 191
Peach Stone Fuel; Hypnotism; Myrtol..... ... 192
NOTES AND FORMULÆ.
Perfect Antiseptic; Irritible cough in Phthisis, &c.; Mistura Antifebrilis .......... 193
Anæsthetic Mixtures; Vomiting of Pregnancy; Asthma .......... 194
EDITORIAL.
Is the Code of Ethics a Failure?; The Georgia Medical Association .......... 195
Dr. C. C. Blackburn; I Phillips; American Medical Association .......... 196
Urinary Test Papers; The Medical and Dental Departments of the Sou. Med. College .......... 197
Book Notices .......... 197, 198, 199
Special Notes .......... 199, 200

